# Friend, Foe or Both? Immune Activity in Alzheimer’s Disease

**DOI:** 10.3389/fnagi.2019.00337

**Published:** 2019-12-10

**Authors:** Georgia R. Frost, Lauren A. Jonas, Yue-Ming Li

**Affiliations:** ^1^Chemical Biology Program, Memorial Sloan Kettering Cancer Center, Manhattan, NY, United States; ^2^Pharmacology Program, Weill Cornell Graduate School of Medical Sciences, Cornell University, Ithaca, NY, United States

**Keywords:** neuroinflammation and neurodegeneration, glia, cytokine, amyloid, tau

## Abstract

Alzheimer’s disease (AD) is marked by the presence of amyloid beta (Aβ) plaques, neurofibrillary tangles (NFT), neuronal death and synaptic loss, and inflammation in the brain. AD research has, in large part, been dedicated to the understanding of Aβ and NFT deposition as well as to the pharmacological reduction of these hallmarks. However, recent GWAS data indicates neuroinflammation plays a critical role in AD development, thereby redirecting research efforts toward unveiling the complexities of AD-associated neuroinflammation. It is clear that the innate immune system is intimately associated with AD progression, however, the specific roles of glia and neuroinflammation in AD pathology remain to be described. Moreover, inflammatory processes have largely been painted as detrimental to AD pathology, when in fact, many immune mechanisms such as phagocytosis aid in the reduction of AD pathologies. In this review, we aim to outline the delicate balance between the beneficial and detrimental aspects of immune activation in AD as a more thorough understanding of these processes is critical to development of effective therapeutics for AD.

## Alzheimer’s Disease

Alzheimer’s disease (AD) is the most common neurodegenerative disorder and the 6th leading cause of death in the United States, next to heart disease and cancer (James et al., 2014; Alzheimer’s Association, 2015). Currently, no treatment for AD exists, making the development of therapeutics essential to public health (Katsurabayashi et al., 2016). There are two well-established hallmarks of AD: amyloid beta (Aβ) plaques and neurofibrillary tangles (NFT) (Lue et al., 1996; DeTure and Dickson, 2019). In large part, therapeutic efforts have focused on targeting and reducing the accumulation of Aβ and NFT (Panza et al., 2009). However, recent genome-wide association studies (GWAS) indicate that a large percentage of AD risk genes are associated with innate immunity and inflammation, suggesting that the immune system plays a critical, and previously unappreciated, role in AD pathology (Bis et al., 2018; Pimenova et al., 2018).

Clinical observations and bioinformatics have demonstrated that inflammatory cytokines, chemokines and other mediators are upregulated in AD patients, which further emphasizes the involvement of the immune system and neuroinflammation in AD (Tarkowski et al., 2003; Cribbs et al., 2012; Zhang et al., 2013; Brosseron et al., 2014). Neuroinflammation is a broad term used to describe the innate immune response to CNS tissue damage. Neuroinflammation occurs when injury, infection, or disease (in the case of AD, misfolded proteins, namely Aβ and NFT) stimulates resident cells to produce inflammatory mediators such as cytokines and chemokines, but also, prostaglandins, free radicals, complement factors, and adhesion molecules which recruit and activate additional immune cells (Calsolaro and Edison, 2016; Schain and Kreisl, 2017; Shabab et al., 2017). Neuroinflammation also involves the proliferation and activation of glial cells, astrocytes and microglia, as well as the presence of the aforementioned inflammatory mediators in the brain (Shabab et al., 2017). Many different cells within the CNS, including microglia and astrocytes, but also, neurons and endothelial cells, are capable of initiating an inflammatory response (Schwartz et al., 2013).

The CNS innate immune response is poorly understood and research suggests there are many differences in comparison to the peripheral immune system (although, alterations in the peripheral immune system have also been implicated in AD progression) (Cao and Zheng, 2018). Notably, immune associated clearance and repair pathways face greater challenges within the CNS environment, such as the presence of the blood brain barrier and ineffective clearance, as well as the abundance of post-mitotic cells (i.e., neurons) and the inability for growth and repair (Herrup and Yang, 2007; Muldoon et al., 2013). Therefore, it is critical to develop a better understanding of the brain’s immune response in neurodegenerative disease and in AD.

While there is consensus that the immune system is intimately involved in AD, there is considerable debate over which aspects of inflammation are harmful and contribute to degeneration, and which are protective and may prevent cognitive decline. Furthermore, it has yet to be established which components of the immune system actively play a role in pathology and which are just a consequence of disease. As mentioned, gliosis, or increased numbers of activated astrocytes and microglia are a hallmark feature of neuroinflammation. However, past descriptions of this phenomenon, namely just “reactive” or “increased gliosis” are vastly oversimplified (Sofroniew and Vinters, 2010). Recent evidence highlights altered glia-specific pathways in post-mortem AD tissue and in mouse models of AD, suggesting that glial responses are much more heterogeneous and complex than previously thought (Thome et al., 2018). Overall, the broad spectrum of glial changes and functional consequences of gliosis in AD are not yet understood.

While neuroinflammation can promote efficient clearance of Aβ and neuronal debris it can also accelerate disease by causing neuronal and glial cell death (Qin et al., 2002; Block et al., 2007). This inflammatory balance is highly orchestrated and understanding how to regulate these responses is key to developing effective therapeutics for AD. The initiation of an immunological reaction can be beneficial and critical, allowing for a burst of glial activity to protect and repair the site of damage, and to clear toxic species or dysfunctional synapses (Wang M. M. et al., 2018). For example, in response to adverse conditions, microglia will undergo morphological changes, accompanied by the release of a storm of molecular mediators that increases clearances of Aβ (Sole-Domenech et al., 2016; Zhao et al., 2017). Furthermore, various types of non-neuronal cells are recruited to the site to assist in repairing the damage and consolidating excessive inflammation (Sofroniew, 2015). These reparative processes are beneficial, yet may also have harmful consequences such as sustained cytokine release which can become toxic to neuronal cells (Heneka et al., 2015). Therefore, understanding the specific cellular roles and inflammatory reactions in AD is of vital importance.

**FIGURE 1:**
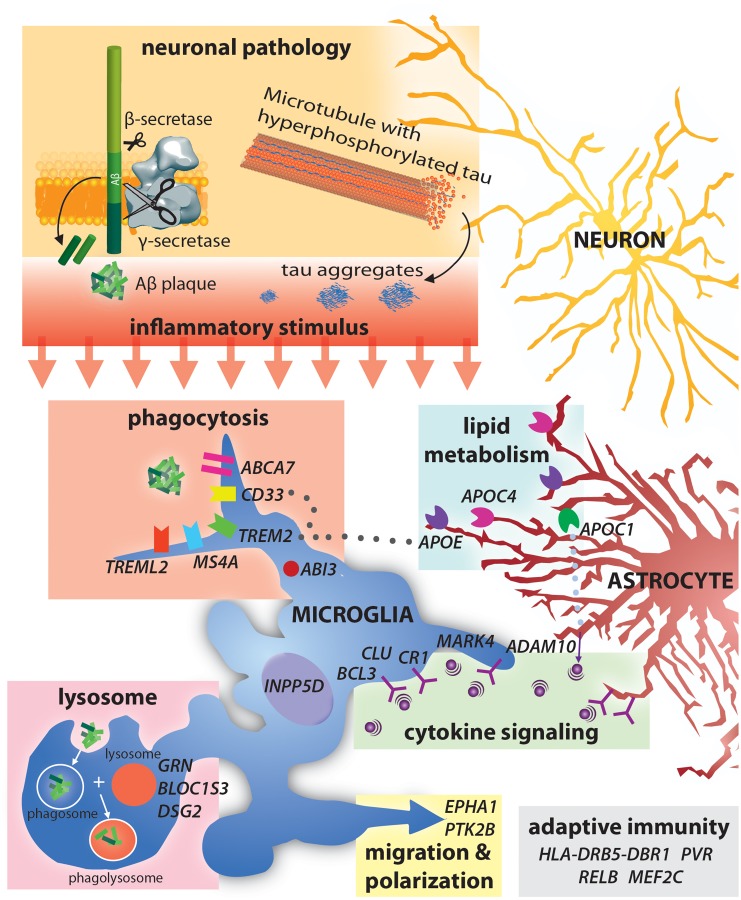
Alzheimer’s disease risk-loci are involved in a variety of immune functions including phagocytosis and lysocytic activity, cytokine signaling and adaptive immunity. Neuronal production of Aβ and tau aggregates may be the initial trigger for this immune activity in AD.

While studying familial Alzheimer’s disease (FAD) mutations has provided insight into disease etiology, FAD accounts for only a small proportion of all AD cases (St George-Hyslop et al., 1989). Sporadic Alzheimer’s disease (SAD) is a late onset, multi-factorial disease for which the biggest risk factor is age (Dorszewska et al., 2016). However, genetic predisposition still plays a considerable role in risk of developing SAD with a heritability estimate of 60–80% (Gatz et al., 2006). Linkage studies have highlighted genes involved in AD pathogenesis beyond those directly involved in Aβ production identified in FAD studies (Bertram et al., 2008). FAD mutations are associated with Mendelian patterns of inheritance and age-dependent penetrance. These genes cause AD but are extremely rare in the general population (Tanzi, 2012). In contrast, genes identified in SAD linkage studies have higher prevalence in the general population but confer a much smaller risk of developing AD (Figure 1; Pimenova et al., 2018; Naj et al., 2011). Understanding the role that these AD associated genes play will give further insight into AD pathology and possibly result in the identification or novel targets for therapeutic development.

The identification of SAD associated genes has already provided novel insight into pathways involved in AD pathology. AD associated genes can be loosely grouped into those involved in Aβ accumulation, NFT formation and spread, cholesterol homeostasis, innate immunity and inflammation, endocytosis, the cytoskeleton, and epigenetics (Karch and Goate, 2015; Van Acker et al., 2019). Interestingly, many AD associated genes are involved in neuroinflammation and immune activation (Figure 1). Currently, AD associated inflammatory genes such as Trem2 and CD33 are perhaps the most widely discussed aspects of AD pathology. While the specific mechanisms as to how variants in these genes contribute to AD is still up for debate, an established feature of all three is activation of immune function. Notably, AD risk variants in triggering receptor expressed on myeloid cells 2 (TREM2) (Guerreiro et al., 2013; Jonsson et al., 2013) and myeloid cell surface antigen CD33 (Bradshaw et al., 2013) particularly highlight the importance of immune activation in clearing Aβ from the brain (Efthymiou and Goate, 2017).

## Innate Immunity in AD

The original amyloid cascade hypothesis notes that immune system activation occurs after Aβ deposition. However, clinical studies suggest that inflammatory changes occur much earlier in disease than originally thought (Tarkowski et al., 2003; Brosseron et al., 2014). In fact, systemic immune challenge in mice with Polyriboinosinic-polyribocytidylic acid (polyI:C), a dsRNA analog, resulted in an AD phenotype including Aβ plaques, NFT and gliosis, suggesting immune challenge can trigger an AD phenotype (Krstic et al., 2012). Furthermore, a plethora of studies have demonstrated that modulating immune system specific proteins impacts the development of AD pathology in various experimental systems, suggesting that the immune system directly impacts pathology. Both systemic inflammatory conditions, such as obesity and diabetes, and activation of the immune system in the CNS, for example, traumatic brain injury, have been associated with the development of AD (Whitmer et al., 2008; Sivanandam and Thakur, 2012; Qizilbash et al., 2015).

The innate immune system is activated when cells expressing specialized pattern recognition receptors (PRRs) recognize pathogen-associated molecular patterns (PAMPs) on invading pathogens (Akira et al., 2006; Suresh and Mosser, 2013). Host derived PRR ligands, known as danger-associated molecular patterns (DAMPs) will also trigger an immune response. In the case of AD, misfolded or aggregated protein, such as Aβ plaques or NFT are DAMPs that trigger neuroinflammation. Activation of PRR and DAMPs provide the cell with differential signaling outcomes, most likely due to the need to differentiate between pathogen-induced toll-like receptor (TLR) activation, which requires immune participation, and tissue damage-induced TLR activation, which requires a balance between immune intervention and tissue damage repair (Miyake, 2007). PRRs such as TLRs, Nucleotide-binding oligomerization domain, Leucine rich Repeat and Pyrin domain containing (NLRP)s, CD36 and RAGE mediate cellular activation by DAMP’s (Cribbs et al., 2012). This activation leads to transcriptional transduction of genes promoting NFkB dependent proinflammatory gene transcription and NLRP3 inflammasome assembly, ultimately leading to the release of proinflammatory cytokines (Latz et al., 2013; Liu et al., 2017).

## AD Associated Proinflammatory Cytokines

Cytokines are a heterogeneous group of multifunctional proteins that can by synthesized and secreted by almost all cells (Feghali and Wright, 1997). They generally act locally in either a paracrine or autocrine manner (Zhang and An, 2007). Many cytokines are classified as interleukins (ILs) as they are secreted by, and act on, leukocytes. Other types of cytokines are tumor necrosis factors (TNFs), transforming growth factors (TGFs), interferons (IFNs), and chemokines (Feghali and Wright, 1997). Cytokine signaling is involved in a variety of cellular processes including: cell proliferation, gliogenesis and neurogenesis, cell migration, apoptosis, and neurotransmitter release (Boulanger, 2009; Borsini et al., 2015). Chemokines, while considered cytokines, function differently than ILs, TNFs, TGFs, and IFNs, by attracting both adaptive and innate immune cells to the site of insult.

Cytokines are often categorized as either pro inflammatory or anti-inflammatory. The balance between pro- and anti-inflammatory cytokines acts to successfully eliminate a pathogen, while also protecting surrounding tissue from excessive damage (Szelényi, 2001). Imbalance between pro- and anti-inflammatory cytokines could contribute to the development of AD. Cytokines function by binding to their receptors expressed on the surface of a variety of cell types. Expression of these receptors is tightly regulated, both spatially and temporally, as a means of controlling an inflammatory reaction (Szelényi, 2001). Research into the prevalence and function of cytokines in the AD is inconsistent, however, at least 23 cytokine polymorphisms in 13 different cytokines have been associated with risk of developing AD (Zheng C. et al., 2016; Table 1). Of these, studies have demonstrated that IL-1β, IL-6, IL-10, IL-18, IFN-γ, and TNF-α have altered expression in AD (Griffin et al., 1989; van der Wal et al., 1993; Hull et al., 1996). Furthermore, polymorphisms in IL-4, IL-12, and IL-23 and have been associated with AD but there is no consensus on whether their expression is changed with disease. Even though IL-10, IL-1ra and TGF-β are not genetically linked with AD, they have been found at higher levels in AD patients (Zheng C. et al., 2016).

**TABLE 1:** Summary of the involvement of cytokines that play well established roles in AD pathology.

**Cytokine**	**Involvement in AD pathology**
TNF-α	TNF-α is involved in inducing acute phase inflammation and is elevated in AD serum, cerebrospinal fluid (CSF) and cortex (Tarkowski et al., 1999, 2003). Anti-TNF-α treatment reduces Aβ deposition, behavioral impairments, and inflammation in AD animal models suggesting TNF-α is detrimental factor in AD (Russo et al., 2012; Tweedie et al., 2012; Detrait et al., 2014; Gabbita et al., 2015). However, one study suggests that TNF-α expression in APP transgenic mice at early stage induces glial uptake of Aβ (Chakrabarty et al., 2011). Additionally, neuronal TNF-α expression in 3xTg AD mice promotes neuronal death (Janelsins et al., 2008).
IL-1β	IL-1β is rapidly secreted in response to injury and is an important mediator of inflammatory response as well as cell proliferation, differentiation and apoptosis. IL-1β has been found at high levels near the sites of Aβ plaques (Griffin et al., 1989; Das and Potter, 1995; Licastro et al., 2000). Overexpression of IL-1β in APP/PS1 mice activates a phagocytic population of microglia and reduces Aβ plaques (Ghosh et al., 2013; Cherry et al., 2015). Furthermore, mice deficient in the receptor for IL-1β have lower recruitment of microglia to Aβ plaques, supporting the idea that IL-1β can mediate microglial chemotaxis (Kamphuis et al., 2012).
IL-6	IL-6 may be both proinflammatory and anti-inflammatory and is elevated in the plasma, CSF, and brain of AD patients (Ershler and Keller, 2000; Licastro et al., 2000, 2003; Shibata et al., 2002; Baranowska-Bik et al., 2008; Galimberti et al., 2008).
IL-10	IL-10, also known as cytokine synthesis inhibitory factor (CSIF), is an anti-inflammatory cytokine upregulated in AD patients (Guillot-Sestier et al., 2015). Overexpression of IL-10 in AD animal models reduces microglial phagocytosis of Aβ leading to cognitive impairment (Chakrabarty et al., 2015; Guillot-Sestier et al., 2015).
TGF-β	TGF-β is an immunosuppressive cytokine that protects neurons against damage. A genetic polymorphism in in TGFB1 is associated with the risk of developing AD (Luedecking et al., 2000). Post mortem AD brains contain increased levels, specifically in Aβ plaques (van der Wal et al., 1993; Chao et al., 1994). Long-term overexpression of TGF-β by astrocytes can increase Aβ clearance by microglia and improve cognitive impairment (Wyss-Coray et al., 2001; Chen et al., 2015). Conversely, TGF-β induces astrocyte aggregation and Aβ deposition near brain microvessels (Ueberham et al., 2005).
IFN-γ	IFN-γ is a proinflammatory regulatory cytokine that activates microglia. It is primarily produced by T cells and natural killer cells but can also be secreted by microglia and astrocytes (Fultz et al., 1993). IFN-γ is upregulated in the AD brain (Huberman et al., 1994) and a polymorphism is associated with fast progressing AD, suggesting it could play a detrimental role in the course of AD (Asselineau et al., 2015).

## Interferons (IFNs) in AD

Termed for their ability to interfere with viral replication, IFNs are a family of pleiotropic cytokines with broad immunomodulatory functions (Lindenmann, 1982; de Weerd et al., 2013). IFNs are classified based on their cognate receptor: type-I IFN; 14 subtypes, notably IFNα and IFNβ, type-II IFN; IFN-γ, and type-III IFNs; contains three IFNλ subtypes (de Weerd et al., 2013). The three subtypes share pro-inflammatory properties and activate multiple signaling pathways. The most critical of these pathways is the janus-associated kinase (JAK) and signal transducer and activator of transcription (STAT) pathway (JAK-STAT) (Platanias, 2005).

Type-I IFNs signal through their cognate receptor: IFNα/β receptor (IFNAR) which is composed of two subunits, IFNAR1 and IFNAR2 (de Weerd et al., 2013). Upon binding the two subunits recruit JAKs (Tyk-2 for IFNAR1 and Jak1 for IFNAR2) which act as docking sites for the phosphorylation of STATs. Upon phosphorylation, STATs translocate to the nucleus and induce transcription of interferon-regulated genes (IRGs) (Platanias, 2005). Additionally, IFN-regulated factors (IRFs) may bind to STATs prior to nuclear translocation and further modify the IFN response (Honda et al., 2005). Type-I IFN signaling alone may result in regulation of over 2000 genes known collectively as interferon-stimulated genes (ISGs) (Schreiber and Piehler, 2015) – much is still unknown about the downstream outcomes of type-I IFN signaling (Ng et al., 2016).

The functional consequence of IFN signaling within the CNS is particularly diverse and is important for processes such as cell survival and immunomodulation (Gough et al., 2012). IFNα and IFNβ are used therapeutically in multiple sclerosis to suppress autoimmune attacks on myelination (Wingerchuk and Carter, 2014). Furthermore, IFNβ is a driver of microglial phagocytosis of myelin in a murine autoimmune encephalitis model (Kocur et al., 2015). However, an epidemiological study suggests IFNβ treated MS patients develop Parkinson’s disease (PD)-like symptoms, such as impaired motor function and cognitive deficits, with long term type-I IFN therapy (Manouchehrinia and Constantinescu, 2012). While IFN signaling may be beneficial, particularly in the periphery, within the CNS, IFNs may also be detrimental and contribute to the development of many neurodegenerative disorders. To highlight this contradiction, IFNβ-/- mice develop Lewy body and PD like symptoms (Ejlerskov et al., 2015), yet, genetic and pharmacological inhibition of type-I IFN signaling is neuroprotective in the MPTP model of PD (Main et al., 2017). The outcome of IFN signaling within the CNS is highly specific to environmental context and requires further study. Interestingly, there is a body of evidence suggesting that IFN signaling contributes to cognitive impairments in a variety of disease states such as human immunodeficiency virus (HIV) encephalopathy (Rho et al., 1995), West Nile virus (Cunningham et al., 2007), neonatal Aicardi–Goutières syndrome (Crow et al., 2006a, b), and even normal aging (Baruch et al., 2014; Zheng et al., 2014).

The staggering diversity of cytokines and chemokines implicated in the progression of AD pathology serves to highlight the complexity of immune activation in disease. Due to the influence of so many different proinflammatory and anti-inflammatory (or both) mediators in AD, it seems unlikely that targeting any specific cytokine, without fully understanding the downstream effect on the immune system as a whole, will be an effective treatment for AD. Furthermore, cytokines may elicit different effects depending on the cell type and cellular context so the relationship between different cell types in AD needs to be explored.

## Glia

There are many different glial cell types: microglia, astrocytes, oligodendrocytes, ependymal and satellite cells. Until recently, glia were categorized as support cells for neuronal activity, however, accumulating evidence suggests glia have roles that expand far beyond neuronal support. Microglia, the most abundant mononuclear phagocyte in the CNS, are most studied for their role in immune surveillance, phagocytosis, neurodevelopment and synaptic plasticity, while astrocytes provide protection, support, and nutrients to neurons and to the vasculature (Allen and Eroglu, 2017; Li and Barres, 2018). Oligodendrocytes form myelin that coat nerve cell axons, aiding in the propagation of action potentials, while Schwann cells in the PNS act analogously (Lassmann, 1998). Ependymal cells line CNS ventricles and the central canal of the spinal cord and are responsible for the regulation of CSF (Johanson et al., 2008). It is well-established that neurons, astrocytes and microglia all play vital roles in the tripartite synapse and therefore are all necessary for synaptic transmission, learning and memory (Araque et al., 1999). A plethora of evidence highlights the roles of glia in immune activity in AD including phagocytosis of cellular debris and cytokine release (Leyns and Holtzman, 2017).

## Microglia

The CNS contains microglia, resident immune cells which migrate to the brain during embryonic days 8–9 (Hanisch and Kettenmann, 2007; Quan and Banks, 2007; Ginhoux and Prinz, 2015; Louveau et al., 2015; Matcovitch-Natan et al., 2016). Microglia are found throughout the CNS and their immune activity within the brain is well defined (Lawson et al., 1990; Hanisch and Kettenmann, 2007; Ransohoff and Cardona, 2010; Kierdorf and Prinz, 2013; Jay et al., 2015; Lauro et al., 2015; Matcovitch-Natan et al., 2016). Microglia play a central role in the immune system and have receptors for neurotransmitters and hormones, cytokines and chemokines, and PRRs and therefore respond to a variety of inflammatory mediators that are implicated in AD, including Aβ (Liu et al., 2005; Smith et al., 2012; Dewapriya et al., 2013; Rangaraju et al., 2018a, b). Microglial activation is associated with AD progression: however, whether this is harmful, protective, or, both, remains to be established. Microglia may prevent AD pathology by reducing Aβ accumulation – Aβ can be directly removed from central nervous system circulation by activated microglia (Michaud et al., 2013). Microglia have been shown to recognize and phagocytose dysfunctional synapses (Wake et al., 2009; Tremblay et al., 2010) and stimulate synapse formation by secreting brain-derived neurotrophic factor (BDNF) (Parkhurst et al., 2013). On the other hand, microglial cytokine production has also been associated with neurotoxic effects that contribute to cognitive decline (Kettenmann et al., 2011; Heneka et al., 2015; Heppner et al., 2015; Calsolaro and Edison, 2016).

Microglia have a spectrum of phenotypes ranging from ameboid to ramified (Stence et al., 2001; Fontainhas et al., 2011) which are influenced by environmental factors such as the presence of cytokines and chemokines (Ginhoux et al., 2013). On one side of the spectrum, ramified microglia have many processes that facilitate the interaction with nearby neurons, astrocytes and blood vessels. These surveying interactions are necessary for CNS tissue maintenance and neuronal function (Wake et al., 2009; Paolicelli et al., 2011; Schafer et al., 2012; Parkhurst et al., 2013). On the other hand, amoeboid microglia have retracted processes and therefore, the highest level of motility and greatest capacity for phagocytosis (Karperien et al., 2013).

The importance of microglial phagocytosis in neuroprotection and maintaining homeostasis is well appreciated, however, the contribution of dysregulated phagocytosis to disease state has only recently emerged. In fact, impaired microglial clearance of Aβ is thought to play a more significant role in the development of SAD than aberrant Aβ production (Mawuenyega et al., 2010). A decrease in Aβ clearance has been demonstrated in AD patients compared to age-matched controls suggesting impaired microglia or astrocyte clearance may be a causative factor in AD (Mawuenyega et al., 2010). Phagocytic activity can differ with disease and environmental context, resulting in a wide spectrum of phagocytic responses. The phagocytotic pathway can be described in three steps: 1. find-me, 2. eat-me, and 3. digest-me (Sierra et al., 2013; Wolf et al., 2017). The first step is initiated when specific molecules bind to target-recognizing receptors on the microglia. These receptors can be specific toward signaling molecules on the surface of items to be phagocytosed, such as pathogens, dystrophic neurites or protein aggregates. In fact, different receptors trigger different signaling pathways that stimulate phagosome formation (Arcuri et al., 2017). In the case of AD, targets such as Aβ or NFTs are recognized by TLRs resulting in a proinflammatory cytokine storm associated with release of TNF, IL-1 and NO, while recognition of cell debris or dystrophic neurites by microglial TREM2 receptors is associated with a phagocytic response along with an increase in TGFB and IL10 signaling (Neumann and Takahashi, 2007; Takahashi et al., 2007; Neumann et al., 2009). During the second step, eat-me, the plasma membrane extends and encloses around the target forming a vesicular phagosome. This nascent phagosome subsequently fuses with lysosomes forming a phagolysosome. The third and final step, digest-me, occurs within the phagolysosome as the target is degraded. Following this, byproducts must be either stored or recycled by the phagocytic cell (Flannagan et al., 2012).

Specifically, Aβ triggers an inflammatory response which stimulates microglia to clear it from the brain and this prevents the formation of plaques (Colton et al., 2006; Jekabsone et al., 2006; Morgan, 2006; Zhao et al., 2017). After Aβ is engulfed it is targeted to the autophagic pathway for eventual degradation by the fusion of the autophagosome with a lysosome. Alternatively, reactive astrocytes can uptake Aβ and degrade it via the autophagic/lysosomal pathway. Additionally, Aβ may be cleared from the brain by proteolytic degradation. Aβ can be cleaved by the metalloprotease neprilysin (Takaki et al., 2000), therefore, mice treated with neprilysin inhibitors have increased Aβ load (Marr et al., 2003). Insulin degrading enzyme has also been shown to degrade monomeric Aβ (Vekrellis et al., 2000).

Beyond phagocytosis and the clearance of proteins from the brain, microglia are also essential to synaptic function and make direct contact with synapses and dendritic spines during neuronal activity. It is well-established that microglia play a vital role in synaptic pruning during development, but evidence suggests they contribute to synaptic plasticity in the developed brain by a similar mechanism (Hong et al., 2016; Sominsky et al., 2018). Microglia phagocytosis of synapsis and dendritic spines appears most common in the hippocampus (Paolicelli et al., 2011). In particular, microglial expression of complement proteins and receptors seems to play a critical role in recognizing which synapses to phagocytosis (Schafer et al., 2012). Microglia also remove damaged synapses in a neuroprotective process known as synaptic stripping (Trapp et al., 2007; Yamada et al., 2008). On the other hand, when microglia phagocytose Aβ it activates the NLRP3 inflammasome, leading to caspase-1 activation and IL-1β maturation and release which can be harmful to surrounding tissue (Halle et al., 2008). When NLRP3 and caspase-1 are genetically deleted there is reduced Aβ deposition, decreased IL-1β release and improved cognitive performance in APPPS1 mice (Heneka et al., 2013). Additionally, complement binding may stimulate microglia to engulf synaptic contents in the presence of Aβ (Hong et al., 2016). Therefore, microglial phagocytosis of Aβ may be both beneficial and harmful.

Activated microglia can secrete toxic proinflammatory cytokines (Colonna and Butovsky, 2017; Kinney et al., 2018) and also release mediators that induce astrocytes to secrete a neurotoxic substance (Liddelow et al., 2017) contributing to neurodegeneration. On the other hand, many of the previously discussed cytokines released by microglia, also have physiological roles in synaptic plasticity and neurogenesis (Albensi and Mattson, 2000; Bernardino et al., 2008; Morris et al., 2013). For example, IL-1β, TNF, IL-6. and complement cascade proteins are all involved in memory consolidation (Goshen et al., 2007; Boulanger, 2009; Yirmiya and Goshen, 2011). In conclusion, microglial activity and cytokine release are vital to normal cognitive processes, however, in other contexts these processes can be neurotoxic.

## Astrocytes

Astrocytes are another major glial cell type within the CNS. Unlike microglia, which are known to be highly motile, astrocytes operate within territorial domains, either supporting the vasculature or enveloping neuronal synapses, through processes terminating in the endfoot (Oberheim et al., 2006). This enables astrocytes to exert control over various CNS functions including blood brain barrier regulation, nutrient delivery, ion and metabolite balance, and innate immune regulation (Attwell Buchan et al., 2010; Bazargani and Attwell, 2016). Astrocytic end-feet express Ca^2+^ ion channels and glutamate receptors, allowing for the propagation of calcium currents and the release of gliotransmitters. A pertinent member of the tripartite synapse, astrocytes enable communication with and between neurons (Lind et al., 2018). Specifically, astrocytes can uptake and release neurotransmitters such as glutamate, GABA and ATP, neuromodulators; d-serine and kynurenic acid, as well as growth factors and inflammatory mediators (Malarkey and Parpura, 2008; Choi et al., 2014; Martineau et al., 2014). Further, astrocytes offer protection against CNS injury and are critical for repair of nervous tissue. After injury, astrocytes propagate and become reactive, a process known as astrogliosis. Astrogliosis is an evolutionarily conserved and neuroprotective process that contributes to the isolation of damaged tissue through formation of a glial scars and consolidation.

Astrocytes are key regulators of the brains inflammatory response and are capable of releasing, and responding to, a spectrum of immune mediators (Farina et al., 2007). Astrocytes secrete many of the cytokines known to be upregulated in human AD brain samples and in transgenic mouse models of AD (Benzing et al., 1999; Apelt and Schliebs, 2001; Abbas et al., 2002; Tan et al., 2002), notably: IFNγ, IL-1β, TNFα, IL-6, and TGFβ (Constam et al., 1992; McGeer and McGeer, 1995; Hu et al., 1998; Johnstone et al., 1999) and are capable of secreting Aβ (Frost and Li, 2017). Furthermore, Aβ can stimulate the production and secretion of these cytokines from astrocytes (Craft et al., 2004; White et al., 2005). Similar to microglia, astrocytes undergo substantial changes in response to specific stimulus and may be resting or activated depending on the cellular environment (Mrak, 2012). The number of astrocytes is thought to remain constant throughout AD, however, some cells, particularly those in proximity to Aβ plaques, become reactive, while conversely, large numbers of astrocytes atrophy, a process known as astrodegeneration (Rodríguez et al., 2014).

Depending on the exact immune stimulus, astrocytes undergo a range of molecular and morphological changes (Zamanian et al., 2012). Characteristic of “astrogliosis,” astrocytes exhibit hypertrophy of processes, marked by increased expression of intermediate filament, most notably glial fibrillary acidic protein (GFAP) (Wilhelmsson et al., 2006; Kamphuis et al., 2012). Astrogliosis results in differential gene expression of structural proteins, synaptic modulators, and transcriptional, inflammatory and vascular regulators. Further, specific signaling pathways are associated with regulation of astrogliosis. JAK-STAT3 and NFkB signaling induces astrocyte reactivity and proinflammatory cytokine release (Ceyzeriat et al., 2016), while B1-itegrin-mediated signaling permits procurement of mature, non-reactive astrocytes (Robel et al., 2009) indicating the existance of feedback pathways for regulation of gliosis. Increasing evidence has shown the beneifts of astrogliosis, including excitotoxic glutamate uptake, inflammatory cell containment, and neuroprotection against ocidative stress via glutathione production (Sofroniew and Vinters, 2010).

In addition to their immune function, astrocytes are involved in learning and memory in several critical ways: (1) glutamate reuptake, synthesis and metabolism, (2) GABA recycling, (3) regulation of intracellular calcium, (4) regulation of extracellular K^+^ concentration, (5) release of gliotransmitters, (6) control of blood flow and therefore, glucose supply, and (7) regulation of lactate levels (Halassa et al., 2009; Suzuki et al., 2011; Han et al., 2012; Navarrete et al., 2012; Moraga-Amaro et al., 2014; Sahlender et al., 2014). Furthermore, astrocytes respond to neurotransmitters and can activate hundreds of synapses at once, enabling the synchronization of complete neuronal networks (Ben Haim and Rowitch, 2017). As with microglia, the contribution of astrocytes to normal cognition is undeniable, but further work needs to be done to further understand these mechanisms and how they can contribute to disease.

## Oligodendrocytes

Alzheimer’s disease research has often been restricted to gray matter, however, it’s important to note that abnormalities in white matter have been identified in AD patients. In fact, white matter hyperintensities volume is found to be elevated in FAD patients 20 years prior to the onset of symptoms and can be used to predict incidence of AD and rate of cognitive decline in SAD cases as well (Lee et al., 2018; Nasrabady et al., 2018). White matter tracts of the brain are important for learning and memory and are the site of pronounced neuroinflammation and microgliosis (Raj et al., 2017). Oligodendrocytes are glia responsible for producing the myelin in the white matter regions. In addition to this, oligodendrocytes also produce neurotrophic factors which impact neurite growth and neuronal connectivity (Schwab, 1990), and can respond to immune activity in the brain. In fact, complement reactive oligodendrocytes in association with activated microglia can be found in the white matter of aged brains (Duce et al., 2006). Furthermore, microglia have diverse phenotypes depending of the region of the brain and may be more activated in white matter (Olah et al., 2011).

## Glia Barriers

Damage to the CNS, in the form of injury or disease, results in a multifaceted immune response. The formation of glial barriers, also known as glial scars, is one type of response to injury and is formed by both glial and non-glial cells that surround the site of damage (Adams and Gallo, 2018). This phenomenon is most widely studied in the context of traumatic brain injury. Glial scarring has also been found after incidence of ischemic stroke and in neurodegenerative diseases such as multiple sclerosis (Woodcock and Morganti-Kossmann, 2013).

While many have reported the heterogeneity of glial scar formation across brain region and disease-type, the development of the glial barrier often begins with the proliferation of reactive astrocytes near the damaged tissue, and the subsequent orchestration of astrocytes, oligodendrocyte precursor cells and microglia to build a rigid wall surrounding damage (Sofroniew, 2009; Wanner et al., 2013). Ultimately a barrier is formed, culminating in the existence of two distinct regions: the lesion core, consisting of oligodendrocyte precursor, fibroblasts and macrophages, and the penumbra, containing reactive astrocytes and microglia (Anderson et al., 2014). The induction of glial scar formation is specific and is stimulated by a range of signaling molecules including Il-1, IL-6, TGF-B1 EGF, BMPs and LIF, from damaged tissue (Wang H. et al., 2018). These signaling molecules aid in the activation of transcription factors that regulate the astrocytic response to tissue damage. Specifically, signaling for the activation of transcription factors STAT3, SP1, OLIG2, Smad3, and NFkB pathways initiate glial scarring mechanisms including astrocytic hypertrophy, migration, proliferation, gliogenesis, and inflammation (Justicia et al., 2000; Hamby et al., 2012).

A defining feature of senile plaques is the presence of surrounding astrocytes and microglia (Itagaki et al., 1989), in fact, the presence of reactive astrocytes follows amyloid deposition throughout the course of AD (Vehmas et al., 2003). Glial barriers surrounding plaques are neuroprotective in two ways: (1) glia, particularly microglia, can break down and phagocytose amyloid plaques, (2) astrocytes can prevent the spread of toxic material and therefore, limit collateral damage (Faulkner et al., 2004) (Anderson et al., 2016). Studies have shown that a deficiency in the ability to form glial barriers worsens AD pathology. When GFAP was knocked out in a mouse model of AD there was a marked increase in plaque-associated dystrophic neurites (Kraft et al., 2013). Further, research shows that glial barriers can aid in axonal regrowth and repair in brain regions where barrier stiffness impacts neuronal growth are more widely affected (Yu and Bellamkonda, 2001; Bollmann et al., 2015).

Glial barriers surrounding amyloid plaques and NFT-associated damage may initially aid in the breakdown and consolidation of toxic debris from the CNS yet subsequently limit the brain’s ability to reform neuronal connections. Moreover, neurons are post mitotic and are unable to divide, making recovery from glial scarring even more difficult (Herrup and Yang, 2007). Thus, immune therapies aimed at regulating the formation of glial scars in AD could help to promote the beneficial regrowth of neuronal connections in the CNS.

## AD Associated Genes

The discovery of immune related AD associated genes from AD linkage studies has provided substantial insight into the involvement of immunity in AD pathology. Numerous variants in immune related genes have been identified in whole-genome sequencing and GWAS analyses, including APOE, BIN1, CD33, CLU, CR1, INPP5D, PICALM, PLCG2, MS4A6D, and TREM2, these genes are involved in a wide range of immune related functions (Table 2; Figure 1; Efthymiou and Goate, 2017; Long and Holtzman, 2019; McQuade and Blurton-Jones, 2019; van der Lee et al., 2019). While most of the identified loci confer only a small contribution to AD development, they serve to highlight the involvement of various immune functions in the development of AD. A notable example is the Apoliprotein E (APOE) gene. APOE has three alleles (e2, e3, e4) accounting for proteins that differ by only one or two amino acids (Ward A. et al., 2012; Najm et al., 2019). A single copy of APOE4 increases risk of developing AD by 4-fold, and individuals homozygous for APOE4 have an approximately 12-fold increased risk of AD (Bu, 2009). The APOE4 allele is more common than FAD mutations and also has a substantial effect on risk of developing AD (Corder et al., 1993). Conversely, the e2 allele may protect against developing AD (Berlau et al., 2009). Post mortem analysis revealed that both AD patients and healthy controls with the APOEe2e3 genotype have reduced amyloid load (Lippa et al., 1997). A possible mechanism behind this phenomenon is that APOE allele can affect the efficiency of Aβ clearance (Kim et al., 2009) and also impact the formation of and morphology of NFT (Shi et al., 2017). The C→A substitution at position 158 allows for differential lipidation and higher APOE2 stability and resistance to thermal and chemical denaturation (Morrow et al., 2000; Ma et al., 2018). Thus, differential physical interactions and binding between APOE and soluble Aβ may be the cause for this change in clearance efficiency. Since presence of the e4 allele is thought to reduce Aβ clearance, this underscores the importance of immune clearance of toxic proteins from the brain in preventing AD symptoms. In fact, APOE alleles have been found to impact the efficacy of passive anti-Aβ immunization suggesting that the different alleles effect microglia’s phagocytic ability for Aβ (Pankiewicz et al., 2017). Many other SAD associated genes have also been shown to play vital roles in immune reactivity in the brain.

**TABLE 2:** Alzheimer’s disease risk-loci with proposed immune functions.

**SNP ID**	**Proposed gene**	**Immune function**
**Cytokine signaling**		
rs2305421	ADAM10	Cleaves TNFα (Mezyk-Kopec et al., 2009)
rs679515, rs3818361	CR1	Complement receptor, Aβ clearance (Rogers et al., 2006)
rs2965101, rs2927438	BCL3	Regulator of NF-kB (immune cell survival and inflammatory response) (Poveda et al., 2017)
rs11136000	CLU	Inhibition of complement system, lipid transport, cell survival (Foster et al., 2019)
rs8100183	MARK4	Limits inflammasome (Li et al., 2017)
rs4420638	APOC1	Inhibits pro-inflammatory cytokine secretion from astrocytes and microglia (Cudaback et al., 2012)
**Phagocytosis and microglial function**		
rs3865444, rs3826656	CD33	Phagocytosis (Griciuc et al., 2013)
rs558678, rs554311 rs610932, rs11824773 rs10897011, rs7926729 rs610932, rs983392	MS4A2 MS4A4A MS4A4E MS4A6A	*Immune* and complement systems regulation and phagocytosis, TREM2 regulation (Ma et al., 2015; Deming et al., 2019)
rs75932628	TREM2	Phagocytosis, microglia migration and activation (Gratuze et al., 2018)
rs9381040	TREML2	Immune activation, phagocytosis (Zheng H. et al., 2016)
rs3851179, rs541458	PICALM	Endocytosis and Aβ clearance (Zhao et al., 2015)
rs3764650, rs3752246	ABCA7	Mediates phagocytic, involved in microglial Aβ clearance (Aikawa et al., 2018)
rs616338	ABI3	Microglial function, actin polymerization (Satoh et al., 2017)
rs35349669	INPP5D	Microglia function and survival (Efthymiou and Goate, 2017)
**Lysosomes**		
rs597668	BLOC1S3	Lysosome biogenesis (Zhang et al., 2014)
rs8093731	DSG2	Lysosomal function (Karch and Goate, 2015)
rs5848	GRN	Lysosomal function (Paushter et al., 2018)
**Astrocytes and lipid metabolism**		
rs5167	APOC4	Lipid metabolism (Riedel et al., 2016)
rs2075650	APOE	Lipid metabolism, immunomodulation, interacts with TREM2 (Shi and Holtzman, 2018)
**Immune cell movement and migration**		
rs11767557, rs11771145	EPHA1	Immune cell trafficking (Yamazaki et al., 2009)
rs28834970	PTK2B	Inflammation, microglia polarization (Okigaki et al., 2003)
**Adaptive immune system**		
rs9271192	HLA-DRB5-DBR1	Antigen presentation (Karch and Goate, 2015)
rs2301275	PVR	NK and T cell function (Stamm et al., 2018a, b)
rs2376866 rs117612135	RELB	Dendritic cell differentiation, regulation of adaptive immune response (Zanetti et al., 2003)
rs190982	MEF2C	B cell proliferation and antigen presentation (Sao et al., 2018)
		

Rare variants in TREM2, which are thought to be loss of function, and increase the risk of developing AD by approximately 2- to 4-fold, were identified by whole-genome sequencing (Sierra et al., 2013; Wolf et al., 2017). A SNP resulting in an Arg-to-His change at amino acid 47 (R47H) is the best established of these variants (Guerreiro et al., 2013; Jonsson et al., 2013). TREM2 binds to the aforementioned AD associated gene APOE and other apolipoproteins, including APOA1, APOB, and APOJ (Yeh et al., 2016). TREM2 associates with DAP12, upon ligand binding, DAP12 is phosphorylated which leads to the recruitment of spleen tyrosine kinase (Syk). Subsequently, Syk signals through activation of phosphatidylinositol 3-kinase (PI3K), and mitogen-activated protein kinases (MAPKs) and the elevation of intracellular Ca^2+^ through release of IP3-gated Ca^2+^ stores (Colonna and Wang, 2016). A number of cellular functions have been attributed to this signaling, including: inhibition of inflammatory signaling, phagocytosis and cell survival (Colonna, 2003). Furthermore, Aβ has been demonstrated as a TREM2 ligand capable of triggering TREM2 signaling (Zhao et al., 2018; Zhong et al., 2018).

TREM2 mediated phagocytosis is critical for Aβ and neuronal debris clearance in AD (Kleinberger et al., 2014; Xiang et al., 2016; Yeh et al., 2016). Specifically, TREM2 expression is important for microglia to physically associate with Aβ plaques (Ulrich et al., 2014; Wang et al., 2016; Yuan et al., 2016; Jay et al., 2015, 2017a,b). In fact, high-resolution confocal microscopy revealed that microglial processes that are in contact with Aβ have enhanced expression of TREM2 and DAP12, possibly suggesting an enrichment of activated DAP12 signaling. These findings suggest that TREM2 is necessary for sustaining or initiating microgliosis in AD (Yuan et al., 2016). Presence of the *Trem2* R47H allele in an AD mouse model results in reduced TREM2 expression around plaques, a decrease in microglia associated with plaques and an increase in neuritic dystrophy near plaques (Cheng-Hathaway et al., 2018). Additionally, TREM2 is important for microglia survival as TREM2−/− mice have increased apoptosis in plaque-associated microglia (Wang et al., 2016). Furthermore, soluble TREM2 (sTREM2) levels in the CSF are correlated with AD progression (Suarez-Calvet et al., 2019).

Taken together, the critical role of TREM2 in AD underscores the involvement of the immune system, particularly the aspect of microglial activation and phagocytosis, in pathology. Features of immune activation are thus beneficial, as microglia play a critical role in the surveillance and recognition of toxic species, in the initiation of an immune response and in the final clearance and degradation of pathogens.

CD33, a myeloid cell transmembrane receptor, is another top ranked AD associated gene identified by GWAS studies; two main variants, rs3865444 and rs12459419, confer risk of developing AD, and higher expression in the brain has been associated with advanced cognitive decline and AD (Carrasquillo et al., 2011; Hollingworth et al., 2011; Naj et al., 2011; Lambert et al., 2013; Malik et al., 2013; Li et al., 2015; Dos Santos et al., 2017). The rs9865444 variant is associated with increased CD33 expression and microglial activation with reduced AB42 internalization and increased brain amyloid load, while the rs12459419 variant has been identified as protective. CD33, a sialic-binding immunoglobulin-like lectin (Siglec-3), is most studied for its role in immunoreceptor tyrosine-based inhibitor motif (ITIM) signaling. CD33 signaling works counter to TREM2 signaling; Upon ligand binding to (i.e., sialylated glycolipids or amyloid plaques), CD33 signals for the phosphorylation of ITIM by SHP1/2, resulting in the inhibition of a variety of receptors containing the immunoreceptor-tyrosine-based activation motifs (ITAM). Inhibition of ITAM results in reduced cellular activity and suppression of proinflammatory cytokine release, oxidative burst and phagocytosis (Lajaunias et al., 2005; Jiang et al., 2014). The rs12459419 variant results in altered splicing of the CD33 mRNA and a loss of the sialic acid binding domain, thereby inhibiting ITIM inhibition and preserving the cell’s ability to phagocytose (Malik et al., 2013).

CD33 is elevated in the AD brain in microglia and infiltrating macrophages and is thought to modulate microglial activation and Aβ clearance (Griciuc et al., 2013; Walker et al., 2015). In fact, knock-out of CD33 in AD mouse models results in reduced Aβ plaque burden (Griciuc et al., 2013). Additionally, CD33 inhibits microglial cytokine release and immune cell proliferation (Tateno et al., 2007; Crocker et al., 2012) further supporting the involvement of inflammation and innate immunity in AD etiology. Further, the hypothesis that CD33 inhibition could increase phagocytosis of Aβ has fueled efforts to design immunotherapy agents and small molecule inhibitors for therapeutic use. However, strides to find specific pharmacological inhibitors of CD33 in the brain have been difficult due to the lack of an atomic resolution structure of the sialic binding domain.

## Current Immune Related Therapies

Currently available treatments for AD are limited to cholinesterase inhibitors and memantine, both of which work by modifying transmission of the neurotransmitters: acetylcholine and glutamate, respectively. (Thomas and Grossberg, 2009; Ferreira-Vieira et al., 2016) While these drugs have proven effective for the slowing of symptoms in mild to moderate AD, no current therapies are effective in significantly slowing of AD progression (Burns et al., 1999; Reisberg et al., 2006). Epidemiological evidence has suggested non-steroidal anti-inflammatory drugs (NSAIDS) are beneficial in the slowing of AD progression – individuals taking NSAIDSs for chronic inflammatory conditions have a lower incidence of AD (de Craen et al., 2005). These studies led to a series of clinical trials testing the effects of naproxen or celecoxib on AD progression. Naproxen’s mechanism of action is through reversible inhibition of both COX-1 and COX-2 enzymes, resulting in the reduction of prostaglandin synthesis. Prostaglandins act as inflammatory inducers, and thus naproxen works as a non-selective inhibitor of an inflammatory response (Robinson, 1983). NSAIDS and naproxen, were originally thought to placate AD pathology by targeting the immune system. However, results from the INTREPAD study indicate that naproxen had no clinical benefits as adults that took naproxen for 2 years did not show slowed cognitive impairment or a decrease in AD-CSF markers (Pellicano, 2014).

It is possible that failure of these clinical trials was due to poor study design rather than a lack of efficacy. The majority of these trials took place early in disease progression, a time when inflammation is critical to initial clearance and consolidation. Therefore, while these studies deem NSAIDS not effective in preventing AD development, targeting inflammation remains a feasible goal. Many studies illustrate the ability to slow cognitive decline with a more selective reduction of detrimental inflammation in AD, while maintaining repair mechanisms. Thus, while studies for the use of NSAIDS in AD proved less than favorable, accumulated research supports the testing of immune modifying therapies.

Immunotherapy has emerged as one of the most promising strategies to tackle AD as it would enable specific clearance of the accumulated toxic proteins of Aβ and NFT without effective other functions in the brain (Valera and Masliah, 2013). Specifically, the development of active and passive vaccines against Aβ, has been utilized as a therapeutic approach (Folch et al., 2016). Active immunotherapy exploits the body’s own immune system and therefore is longer lasting, however, has a high risk of triggering an autoimmune reaction. Furthermore, active immunotherapy can be limited by epitope availability and specificity (Bard et al., 2003; Winblad et al., 2014). Alternatively, passive immunotherapy can be far more specific but has a short effect and therefore requires continuous antibody treatment (Ward M. et al., 2012).

One of the earliest Aβ immunotherapy trials utilized AN1792, a synthetic Aβ peptide, that induces the generation of antibodies against Aβ by active immunotherapy. Unfortunately, 6% of patients during phase 2 of clinical trials developed meningoencephalitis (Gilman et al., 2005; Cao et al., 2018). Furthermore, treatment did not provide a protective cognitive effect despite significantly reducing Aβ pathology. However, a follow-up study performed 4 years after immunization found improvements in cognition in 68% of patients. reduction in Aβ plaque load locally in the brain. Interestingly, a follow-up study, done 3.6–4.6 years after immunization with AN1792, found significant improvements in brain volume and cognitive decline in immunized vs. placebo group. Although, these improvements were only observed in 68% of antibody responders which was less than 20% of the total patients immunized (Vellas et al., 2009). Another active immunotherapy CAD106 has been found to be safe and well tolerated in clinical trials and is still in development (Farlow et al., 2015).

Two notable passive immunotherapeutic agents have entered clinical trials: bapineuzumab and solanezumab. Bapineuzumab that targets the *N*-terminus of Aβ and failed clinical trials due to a poor safety profile in mild to moderate AD patients (though some of these were APOE4 carriers) (Doody et al., 2014; Liu et al., 2015; Vandenberghe et al., 2016). Solanezumab is a humanized IgG1 monoclonal antibody targeting soluble monomeric Aβ that has passed Phases 1 and 2 of clinical trials with a favorable safety profile. Unfortunately, solanezumab did not show efficacy in phase 3 and was abandoned until it reentered clinical trials for FAD patients (Doody et al., 2014; Siemers et al., 2016).

Aducanumab is also a monoclonal antibody. In August 2016, very promising phase I results were published showing that Aducanumab decreased soluble and fibrillar forms of Aβ and slowed down cognitive decline 54 weeks after treatment in a dose-dependent manner (Sevigny et al., 2016). However, phase 3 trials for Aducanumab were canceled in March of 2019 after an independent data monitoring committee concluded that the trial would not meet its primary end points. Despite this, in October 2019, Biogen Inc., announced that they would resume clinical trials following further analysis of the phase 3 data (Biogen, 2019).

## Infection Hypothesis

The antimicrobial peptide (AMP) hypothesis of AD suggests that Aβ is initially generated as a defense mechanism against a real or perceived infection signifying a direct link between AD and innate immunity (Moir et al., 2018). AMPs are evolutionarily conserved proteins that participate in the innate immune system’s first line of defense, with roles in microbial killing and immune regulation (Haney et al., 2017). AMPs require oligomerization for normal functioning, and soluble oligomers form fibrils that target viruses, fungi, bacteria, and some forms of cancerous host cells (Arnusch et al., 2007; Wiesner and Vilcinskas, 2010). Aβ has structural similarities to a family of host-encoded AMPs and possesses antimicrobial activity. *In vitro* studies show Aβ as having a greater potency compared to the archetypal human AMP LL-37 against microbes (Soscia et al., 2010). Further, *in vivo* and *in vitro* studies demonstrate how AMPs promote amyloid fibrilization for use in disruption of microbial cell membranes, neutralization of bacterial endotoxins and pathogen entrapment (Sood et al., 2008; Wang et al., 2014; Chai et al., 2017). Additionally, Aβ42-overexpressing cells are resistant to killing by yeast and bacteria (Eimer et al., 2018). The Aβ-yeast interaction caused Aβ fibril nucleation allowing for the entrapment of yeast, a mechanism that is comparable to the antimicrobial activity of many AMPs (Eimer et al., 2018). Moreover, a mouse model overexpressing Aβ42 displayed higher resistance to certain yeast and bacterial infections (Soscia et al., 2010). Further, recent reports have indicated higher levels of AMPs and pathogen derived epitopes in post mortem AD brain tissue (Williams et al., 2013; Alonso et al., 2014). Pathogen signatures (i.e., Chlamydia Pneumoniae, HSV-1 DNA) specifically colocalize with AD pathology (Balin et al., 1998; Wozniak et al., 2009). Further, HSV infection is significantly associated with development of AD and HSV-1 is shown to increase Aβ generation (Letenneur et al., 2008).

Amyloid beta was previously deemed physiologically irrelevant, however, the presence of antimicrobial activity supports a role in the innate immune response and thus could be an important finding for AD research. Knowledge of Aβ’s AMP capabilities may help put into perspective Aβ and the immune system’s causative role in Alzheimer’s disease. If the physiological role of APP is in pathogen/virus defense, then an overproduction of Aβ could be the result of an innate immune reaction. Indeed, the AMP hypothesis helps to shed a new light on aspects of immune involvement in AD pathogenesis. Understanding the initial benefits provided by amyloid’s AMP properties could prove beneficial to disease treatment.

## Other Neurodegenerative Diseases

Recent genetic evidence has indicated a critical role for inflammation in many neurodegenerative diseases including Multiple Sclerosis (MS), Huntington’s disease (HD), Parkinson’s disease (PD), Ischemia, traumatic brain injury (TBI), and Parkinson’s disease (PD) (Dzamko et al., 2015; Baranzini and Oksenberg, 2017). A common link between these disorders is chronic activation of the innate immune system through specific activation of resident glial cells, release of inflammatory mediators, and the potential for recruitment of peripheral immune cells. While the specifics of immune activation vary between disorders, due to the pathogenic environment, it is clear that inflammation can play both a beneficial and harmful role in these other neurodegenerative diseases as well. Based on the initial trigger (i.e., alpha-synuclein in PD, ROS in Ischemia) chronic immune signaling can lead to neurotoxicity, increased oxidative stress and synaptic and neuronal death (Stephenson et al., 2018; Hassanzadeh and Rahimmi, 2019). However, immune activation can also be beneficial, resulting in the clearance of cell debris and aggregated/misfolded proteins, in the consolidation of inflammation, and in the mediation of cellular repair.

For example, MS is an autoimmune demyelinating disease of the CNS. While chronic immune activity is responsible for neurodegeneration in MS, evidence has also illustrated that inflammation is necessary for clearance of blood-borne inflammatory cells via emigration or *in situ* apoptosis. Proinflammatory cytokines, such as IFNγ and TNFa, promote *in situ* apoptosis of infiltrating T cells, as well as remyelination. Further, macrophages are critical in phagocytosing myelin debris *in situ* highlighting the beneficial immune activities in MS (Furlan et al., 2001).

Following brain injury, such as TBI and ischemic stroke, microglia actively promote injury−induced neurogenesis via production of insulin−like growth factor−1, which suppresses apoptosis and increases proliferation and differentiation of neural stem cells (Thored et al., 2009). Further, microglial activation aids in the consolidation of injured tissue and resultant inflammation, thereby limiting the spread of damage (Kitamura et al., 2009; Thored et al., 2009; Cunha et al., 2018). During PD, alpha-synuclein activates microglia, resulting in excessive release of pro-inflammatory mediators and an overactivation of the phagocytic pruning pathway on dopamine neurons (De Virgilio et al., 2016). Simultaneously, anti-inflammatory microglia are responsible for cytokine cleanup and phagocytosis of extracellular alpha-synuclein (Ferreira and Romero-Ramos, 2018). While it is clear the neuroinflammation can lead to disease progression, the beneficial aspects of inflammation remain critical to the underlying repair and clearance pathways in disorders of the CNS.

## Conclusion

The genetic, cellular, and molecular changes associated with AD provide strong support for an innate immune contribution to disease pathology (Kim et al., 2018). However, which aspects of this contribution are positive or negative is still up for debate. In this review, we propose that there is a delicate balance involved in the innate immune response to AD, which can provide beneficial or detrimental effects. We suggest that some aspects of inflammation in AD are necessary and beneficial and are capable of restricting or preventing AD pathology. By harnessing the benefits provided by the AD immune reaction, we can work to find successful treatments for AD. For too long researchers have fixated on the negative consequences of microgliosis and astrogliosis, broadly categorizing glial cells as “reactive” or “activated.” However, recent genetic and cellular data has highlighted the extreme heterogeneity of glial cells, and the spectrum on which they function. Furthermore, while we have focused on the involvement of the innate immune system, substantial evidence suggests that the adaptive immune system also plays a role in pathology which is an area requiring further study (Sommer et al., 2017).

**FIGURE 2:**
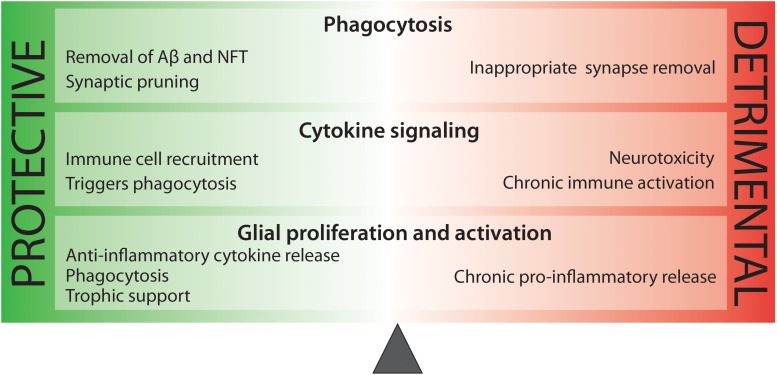
There is a delicate balance between beneficial and harmful immune activity in Alzheimer’s disease. While some aspects of inflammation are protective, such as the phagocytosis of toxic protein species, other processes, such as sustained pro-inflammatory cytokine release are neurotoxic.

While it is clear that inflammation contributes to AD pathogenesis, it is too broad a word to describe the amalgam of innate immune mechanisms that arise during disease. Microgliosis involves phagocytosis of amyloid plaques and dysfunctional synapses, as well as the increased expression of complement proteins for neuronal pruning, and the release of trophic factors for cell plasticity and growth. Conversely, microgliosis increases quantities of cytokine and chemokines which can become toxic and harmful to neuronal cells. Astrogliosis can also be beneficial, resulting in propagation of calcium currents for enhanced signal transduction and enhanced repair and protection. On the other hand, excessive astrogliosis may result in the increase of toxic species. It is very clear that neuroinflammation is heavily involved in AD pathogenesis, but a more precise understanding of the AD-specific immune reaction is critical to our developing effective therapeutics for AD (Figure 2).

## Author Contributions

All authors listed have made a substantial, direct and intellectual contribution to the work, and approved it for publication.

## Conflict of Interest

The authors declare that the research was conducted in the absence of any commercial or financial relationships that could be construed as a potential conflict of interest.
